# Complete Genome Sequence of *Enterococcus durans* Oregon-R-modENCODE Strain BDGP3, a Lactic Acid Bacterium Found in the *Drosophila melanogaster* Gut

**DOI:** 10.1128/genomeA.01041-17

**Published:** 2017-10-05

**Authors:** Kenneth H. Wan, Charles Yu, Soo Park, Ann S. Hammonds, Benjamin W. Booth, Susan E. Celniker

**Affiliations:** Biological Systems and Engineering Division, Lawrence Berkeley National Laboratory, Berkeley, California, USA

## Abstract

*Enterococcus durans* Oregon-R-modENCODE strain BDGP3 was isolated from the *Drosophila melanogaster* gut for functional host-microbe interaction studies. The complete genome is composed of a single circular genome of 2,983,334 bp, with a G+C content of 38%, and a single plasmid of 5,594 bp.

## GENOME ANNOUNCEMENT

*Enterococcus* is part of the large genus of lactic acid bacteria (LAB). In *Drosophila*, a number of species have been identified as symbionts, including *Enterococcus faecalis*, *Enterococcus faecium*, *Enterococcus gallinarum*, and *Enterococcus durans* (1). This report describes the sequence of *E. durans* Oregon-R-modENCODE strain BDGP3, associated with a sequenced *Drosophila melanogaster* host.

*E. durans* Oregon-R-modENCODE strain BDGP3 was isolated from *Drosophila melanogaster* guts. Bacteria were streaked onto brain heart infusion (BHI) agar plates (catalog number 241830; BD Difco). Purified colonies were amplified in liquid BHI (catalog number 211059; Difco) culture overnight, and an aliquot was used for 16S V1 and V4 PCR (2) and sequence identification by ABI 3730XL Sanger sequencing (reviewed in reference 3). Genomic DNA for sequencing was isolated by treating cells with protease, followed by cetyltrimethylammonium bromide (CTAB) extraction (4).

Whole-genome DNA sequencing was performed by the National Center for Genome Resources (NCGR), Santa Fe, NM, using Pacific Biosciences (PacBio, Menlo Park, CA) long-read sequencing on the RSII instrument (5). A single-molecule real-time (SMRT) cell library was constructed with 5 to 10 µg of input DNA using the PacBio 20-kbp protocol. The library was loaded onto one SMRT cell and sequenced using P6 polymerase and C4 chemistry with 6-h movie times. Sequencing yielded a total of 62,492 reads, with a filtered mean read length of 8,747 bp, totaling 546,630,958 bp (>150-fold coverage for the chromosome and >50-fold coverage for the plasmid). The files generated by the PacBio instrument were used for *de novo* assembly constructed using the Hierarchical Genome Assembly Process 2 (HGAP2) protocol from SMRT Analysis version 2.0 (6, 7). This protocol relies on BLASR for alignment (8), the Celera assembler for assembly (7), and Quiver for consensus polishing (6). The final contigs were manually trimmed and reviewed to produce a single circular chromosome and a single plasmid. Annotations of protein-encoding open reading frames and noncoding RNAs (ncRNAs) were predicted using the Rapid Annotation of microbial genomes using Subsystems Technology tool (9) and the GenBank annotation pipeline (10).

The genome sequence is composed of a single circular chromosome (2,983,334 bp), with a predicted overall G+C content of 38%. The chromosomal genome is predicted to contain 2,612 protein-coding genes, 159 pseudogenes, 3 ncRNAs, 6 rRNA operons, 69 tRNA genes, and one transfer-messenger RNA (tmRNA). Of the 2,612 protein-coding genes, 104 are contained within candidate prophages. Our strain contains a single integrated likely prophage and two partial copies (Phage_Finder; Omic Tools). The single copy contains 36,659 bp, and the 2 partial copies contain 13,567 and 16,827 bp. They constitute 2.24% of the genome. Two cornerstone highly conserved prophage proteins are the large terminase subunit and the portal protein (reviewed in reference 11). Only the 36-kb copy contains genes that encode these proteins. In addition, the genome contains a single plasmid, pEdBDGP3A (5,594 bp, 33% G+C content). pEdBDGP3A contains a candidate gene for the plasmid replication initiation protein RepB.

### Accession number(s).

The complete chromosome and plasmid sequences of *Enterococcus durans* Oregon-R-modENCODE strain BDGP3 are deposited in GenBank under the accession numbers CP022930 (chromosome) and CP022931 (plasmid).
